# Effects of Statin Combinations on Zika Virus Infection in Vero Cells

**DOI:** 10.3390/pharmaceutics15010050

**Published:** 2022-12-23

**Authors:** Erica Españo, Jeong-Ki Kim

**Affiliations:** Department of Pharmacy, Korea University College of Pharmacy, Sejong 30019, Republic of Korea

**Keywords:** antiviral, combination therapy, flavivirus, statins, Zika virus

## Abstract

The Zika virus (ZIKV) remains a global health concern. Thus far, no antiviral or vaccine has been approved to prevent or treat ZIKV infection. In a previous study, we found that lipophilic statins can inhibit ZIKV production in Vero cells. These statins appear to have different potencies against ZIKV infection. Here, we determined whether combinations of statins would have synergistic effects to maximize the efficacy of the statins and to reduce potential side effects. Specifically, we used a modified fixed-ratio assay for the combinations of atorvastatin (ATO) or fluvastatin (FLU) with mevastatin (MEV) or simvastatin (SIM). All combinations with MEV tended towards synergy, especially with higher fractions of MEV in the combinations. The ATO + SIM combination tended towards additivity. The FLU + SIM combination also tended towards additivity except for one combination which had the highest fraction of FLU over SIM among the tested combinations. Overall, certain combinations of ATO or FLU with SIM or MEV may be synergistic. More exhaustive combinatorial assays in vitro and in vivo could help define whether combining lipophilic statins would be beneficial and safe for treating ZIKV infections.

## 1. Introduction

The Zika virus (ZIKV) is a mosquito-borne member of the *Flaviviridae* family of enveloped positive-sense single-stranded RNA viruses. The urban/human-to-human transmission cycle of the ZIKV is maintained primarily by *Aedes aegypti* (*Ae. aegypti*) and likely by *Ae. albopictus* [1,2]. Although the current levels of human ZIKV circulation are considered low, increasing global temperatures are believed to drive these two *Aedes* species towards geographic ranges where humans are immunologically naïve to the ZIKV, making the ZIKV a persistent threat [3]. Human ZIKV infections are largely asymptomatic (50–80%), and, in those who develop symptoms, a ZIKV infection generally causes a mild, self-limiting illness [4,5]. However, ZIKV infections have been associated with Guillain-Barré syndrome in adults and congenital Zika syndrome (CZS) in neonates [6,7,8]. ZIKV infections in pregnant women can lead to fetal loss (4–7%) or CZS (5–14%, including the 4–6% incidence of microcephaly) in the newborn [8,9]. Given the threat of ZIKV epidemics, the World Health Organization (WHO) has included the ZIKV in its list of top priority diseases for research and development in emergency contexts [10]. Despite international efforts, there is still no approved vaccine or antiviral against the ZIKV.

In a previous study, we reported that lipophilic statins (atorvastatin, cerivastatin, fluvastatin, lovastatin, mevastatin, and simvastatin) inhibited ZIKV production in vitro [11]. Statins are among the most prescribed antihyperlipidemic medications. Their pharmacophores compete with β-hydroxy β-methylglutaryl-CoA (HMG-CoA) for the binding site of the HMG-CoA reductase (HMGCR), which catalyzes the conversion of HMG-CoA to mevalonate, a rate-limiting step in the mevalonate pathway that leads to the biosynthesis of cholesterol and isoprenoids [12]. Based on our study, these statins have different potencies against ZIKV infection potentially with different targets in the ZIKV life cycle, with atorvastatin (ATO), fluvastatin (FLU), and mevastatin (MEV) targeting early entry events, while simvastatin (SIM) appeared to have more delayed effects. The differences may be due to structural variations. The statins also did not have dramatic effects on a single cycle of infection. Instead, the effects of statins appeared to be compounded over multiple cycles of infection. This would then potentially require higher doses, more frequent administration, or longer durations of treatment for ZIKV infections. Corroborating our findings, other studies have also reported the activity of statins against other flaviviruses, such as the dengue virus (DENV) and the Japanese encephalitis virus (JEV), in vitro and in vivo [13,14,15].

The United States Food and Drug Administration (US FDA) has recently withdrawn their strong recommendation against the use of statins during pregnancy [16]. They have indicated that certain conditions warrant the continuous use of statins during pregnancy; however, this recommendation does not necessarily translate to fully endorsing the use of statins during pregnancy due to their safety concerns. Given that significant morbidities from ZIKV infections occur in fetuses and neonates, protecting fetuses from complications due to ZIKV infections during pregnancy and keeping them safe from the adverse effects of drugs are the goals of anti-ZIKV therapy [17]. Additionally, lipophilic statins have been associated with muscle toxicity collectively called statin-associated muscle symptoms (SAMS), which, in rare severe cases, may require drug discontinuation [18,19]. Thus, it is important to reduce the statin dose requirements to ensure their safety as a treatment for ZIKV infections.

Here, we determined whether active statins (atorvastatin and fluvastatin) had synergistic effects with prodrug statins (mevastatin and simvastatin) through a modified fixed-ratio combination assay. This was performed to determine whether lower doses of these statins could potentially be applied to maximize their effects against ZIKV infection and to minimize the dose-related toxic effects.

## 2. Materials and Methods

### 2.1. Cells, Virus, and Reagents

African green monkey kidney (Vero; ATCC CCL-81) cells were grown in growth medium consisting of Minimal Eagle’s Medium (MEM; Gibco, Carlsbad, CA, USA) supplemented with 10% fetal bovine serum, antimycotic antibiotics (Gibco), and ʟ-glutamine (Gibco). ZIKV (ATCC VR-1838) was propagated in Vero cells using MEM supplemented with 0.3% bovine serum albumin (BSA, Gibco), antimycotic antibiotics, MEM vitamins (Gibco), and ʟ-glutamine (Infection Medium). The ZIKV particles were harvested at 5 days postinfection (dpi), and culture supernatants were stored at −80 °C until use. ZIKV titers were determined through the plaque assay in Vero cells.

Atorvastatin calcium salt trihydrate (ATO), fluvastatin sodium hydrate (FLU), and simvastatin (SIM) were purchased from Sigma–Aldrich (St. Louis, MO, USA). Mevastatin sodium salt (MEV) was purchased from Calbiochem (San Diego, CA, USA). Statin stocks (20 mM) were prepared in DMSO and stored in aliquots at −20 °C. EZ-Cytox was purchased from DoGenBio, Co., Ltd. (Seoul, South Korea).

### 2.2. Fixed-Ratio Combinations

The combinations we tested were ATO + MEV; ATO + SIM; FLU + MEV; and FLU + SIM. The concentrations and ratios of the statins were assigned based on predetermined starting concentrations, where the statins fully rescued Vero cells from ZIKV infections: 8 µM ATO; 3 µM FLU; 12 µM MEV; and 4 µM SIM. Fractional concentrations and combination ratios (statin A: statin B) were assigned based on the maximum starting concentrations: C1—5:0; C2—4:1; C3—3:2; C4—2:3; C5—1:4; and C6—0:5. For convenience, the maximum concentrations of ATO or FLU were always assigned to be C1 (5:0), and those of MEV or SIM were always assigned to be C6 (0:5), such that, for ATO + SIM, C1 (5:0) had 8 µM ATO; C6 (0:5) had 4 µM SIM; C2 (4:1) had 6.4 µM ATO + 0.8 µM SIM; C3 (3:2) had 4.8 µM ATO + 1.6 µM SIM, etc. Combination ratios and starting concentrations are listed in detail in the Results section (Section 3). All statins were diluted in infection medium on the day of use.

### 2.3. Anti-ZIKV Activity of Statin Combinations in Vero Cells

Vero cells were seeded at a density of 1.2 × 10^4^ cells/well into 96-well plates and incubated overnight at 37 °C and 5% CO_2_ in a humidified atmosphere. The following day, the statins were serially diluted to 2 × the desired concentrations in infection medium with 1% DMSO. The cells were washed once with phosphate-buffered saline (PBS), and either 50 µL of infection medium (cytotoxicity groups) or ZIKV in infection medium (multiplicity of infection, MOI, of 0.02; infection groups) was added to the assigned wells. Then, 50 µL of the statin dilution (2×) was added to the assigned wells (*n* = 3 per concentration per group). For the untreated controls, 50 µL of 1% DMSO was added per well for a final concentration of 0.5% DMSO/well. The plates were incubated at 37 °C and 5% CO_2_ in a humidified atmosphere for 5 days. On the fifth day, 10 µL of EZ-Cytox was added per well, and the absorbance at 450 nm was read using a microplate reader. Cell viability was calculated relative to the untreated uninfected Vero cells per assay plate (0.5% DMSO). Two independent experiments were performed for each drug combination.

### 2.4. Determination of Synergy

The half-maximal effective concentrations (EC_50_) were determined using GraphPad Prism 9.0 (GraphPad Software, Inc., San Diego, CA, USA). First, the raw absorbance data of the treated infected cells were normalized to the maximum (100%, untreated uninfected controls) and the minimum (absorbance of the untreated infected controls). The EC_50_ values were then calculated using the nonlinear regression function of GraphPad Prism using the “log(inhibitor) vs. normalized response—Variable slope” option. The EC_50_ values were then used to calculate the fractional inhibitory index (FIC_50_) for each drug per combination based on Equation (1), where A is one of the two drugs in the combination (C1 to C6). The mean EC_50_ values were obtained from the means of 2 independent experiments and were presented with asymmetric confidence intervals as calculated with GraphPad Prism.

(1) $${\mathrm{FIC}}_{50A\;\left(\mathrm{combination}\right)}=\frac{{\mathrm{EC}}_{50A\;\left(\mathrm{combination}\right)}}{{\mathrm{EC}}_{50A\;\left(\mathrm{alone}\right)}}$$

The combinatorial index (CI) was calculated as the sum of the FIC_50_ of the two drugs (Equation (2)). A CI > 1.0 was considered antagonistic or nonbeneficial; CI = 1.0 was additive or nonsynergistic; and CI < 1.0 was considered synergistic. A normalized isobologram was plotted based on FIC_50A_ and FIC_50B_ per assay. In all cases, the CI for C1 and C6 was always equal to 1.0. The FIC_50_ values were calculated per experiment, and the mean ± standard error of the mean (SEM) from 2 independent experiments was obtained. Isobolograms of the FIC_50_ values were constructed based on the mean FIC_50_ values.

(2) $${\mathrm{CI}}_{\mathrm{combination}}={\mathrm{FIC}}_{50A}+{\mathrm{FIC}}_{50B}$$

## 3. Results

### 3.1. Atorvastatin and Mevastatin

**Figure 1:**
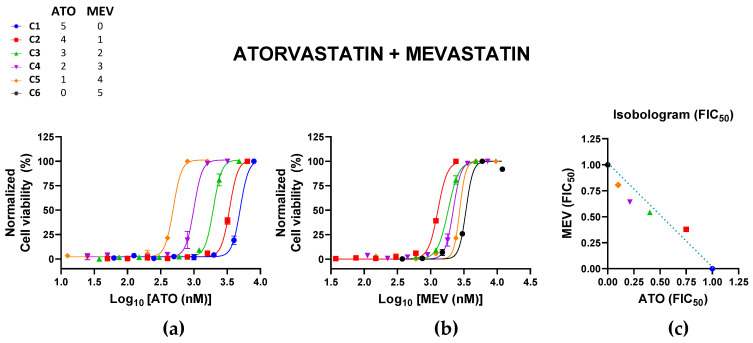
Effects of the combination of atorvastatin (ATO) and mevastatin (MEV) on Zika virus (ZIKV) infection. Vero cells were infected with ZIKV (MOI of 0.02) and treated with different combinations of ATO and MEV. Normalized cell viability of ZIKV-infected cells in response to the different concentrations of (**a**) ATO and (**b**) MEV in their different combinations are shown. All datapoints represent the mean ± SEM of 2 independent experiments per combination. (**c**) The normalized isobologram was constructed based on the mean FIC_50_ of each statin (Table 1). Colors and symbols in the legend correspond to those in (**a**–**c**).

The tested combinations of ATO + MEV were generally additive (CI of 0.90 to 1.00) except for the C4 (2:3) combination from the dilution series based on 3.2 µM ATO + 2.4 µM MEV (Table 1; Figure 1). This combination had a CI of 0.854 (±0.019), which could be considered a tendency towards synergy. All combinations were nontoxic to Vero cells, with cell viabilities > 80% for all combinations (Supplementary Materials, Figure S1a).

### 3.2. Atorvastatin and Simvastatin

**Figure 2:**
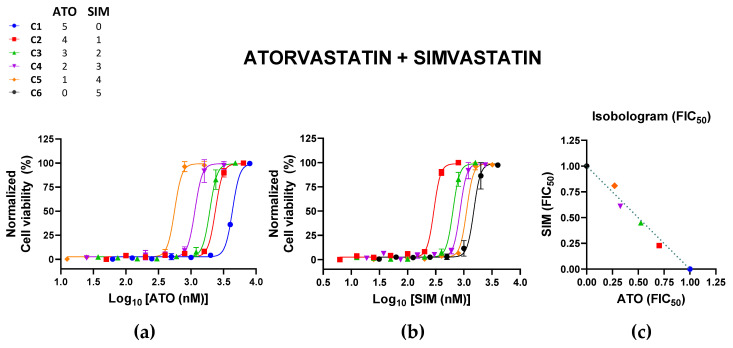
Effects of the combination of atorvastatin (ATO) and simvastatin (SIM) on Zika virus (ZIKV) infection. Vero cells were infected with ZIKV (MOI of 0.02) and treated with different combinations of ATO and SIM. Normalized cell viability of ZIKV-infected cells in response to the different concentrations of (**a**) ATO and (**b**) SIM in their different combinations are shown. All datapoints represent the mean ± SEM of 2 independent experiments per combination. (**c**) The normalized isobologram was constructed based on the mean FIC_50_ of each statin (Table 2). Colors and symbols in the legend correspond to those in (**a**–**c**).

The ATO + SIM C2 to C4 combinations tended towards additivity, with CI values > 0.90 (Table 2; Figure 2). These combinations had similar CI values and large ranges, suggesting that the tested ranges were not significantly synergistic. All combinations were nontoxic to Vero cells, with cell viabilities > 80% for all combinations (Figure S1b).

### 3.3. Fluvastatin and Mevastatin

**Figure 3:**
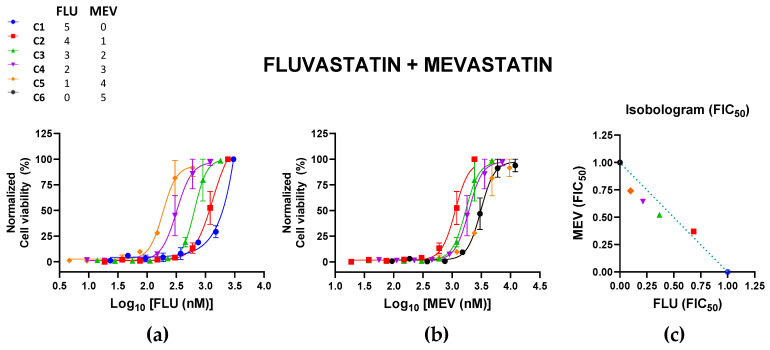
Effects of the combination of fluvastatin (FLU) and mevastatin (MEV) on Zika virus (ZIKV) infections. Vero cells were infected with ZIKV (MOI of 0.02) and treated with different combinations of FLU and MEV. Normalized cell viability of ZIKV-infected cells in response to the different concentrations of (**a**) FLU and (**b**) MEV in these different combinations are shown. All datapoints represent the mean ± SEM of 2 independent experiments per combination. (**c**) The normalized isobologram was constructed based on the mean FIC_50_ of each statin (Table 3). Colors and symbols in the legend correspond to those in (**a**–**c**).

The effects of FLU + MEV against the ZIKV tended towards synergy, with CI values < 0.90 (Table 3; Figure 3); however, considering their broad ranges, these combinations may be primarily additive. The combinations with higher fractions of MEV appeared to have more synergistic tendencies. All combinations were nontoxic to Vero cells, with cell viabilities > 80% for all combinations (Supplementary Materials, Figure S2a).

### 3.4. Fluvastatin and Simvastatin

**Figure 4:**
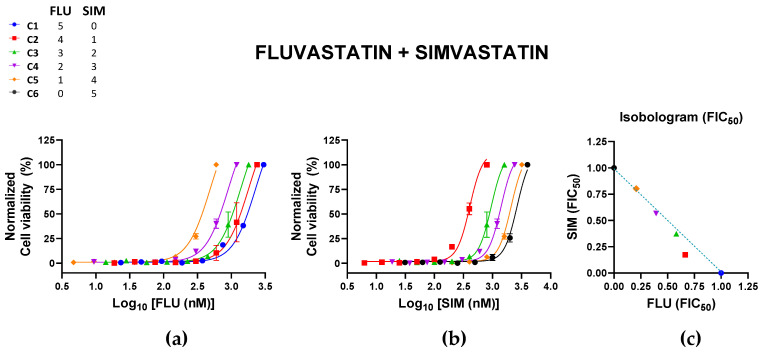
Effects of the combination of fluvastatin (FLU) and mevastatin (SIM) on Zika virus (ZIKV) infection. Vero cells were infected with ZIKV (MOI of 0.02) and treated with different combinations of FLU and SIM. Normalized cell viability of ZIKV-infected cells in response to the different concentrations of (**a**) FLU and (**b**) SIM in these different combinations are shown. All datapoints represent the mean ± SEM of 2 independent experiments per combination. (**c**) The normalized isobologram was constructed based on the mean FIC_50_ of each statin (Table 4). Colors and symbols in the legend correspond to those in (**a**–**c**).

The combination of FLU + SIM also tended towards additivity except for the C2 combination, with a CI value of 0.838 (±0.082) (Table 4; Figure 4). Increasing the fraction of FLU to SIM may be more beneficial to the combination. All combinations were nontoxic to Vero cells, with cell viabilities > 80% for all combinations (Supplementary Materials, Figure S2b).

## 4. Discussion

Owing to the prior strong recommendation of the US FDA against the use of statins during pregnancy, little information on the effects of statins on the human fetus is available. The previous advice against the use of statins in pregnancy was based on animal studies, where statin treatment, specifically atorvastatin and lovastatin (mevinolinic acid), resulted in malformations and low birth weights in rabbits and rats [20,21]. A case report also suggested the possible teratogenic effects of lovastatin [22]. In contrast, small clinical studies on the use of statins during the first trimester of pregnancy and the prescription of statins for the prevention of preeclampsia suggested that, while neonates born to statin users had lower average body weights than those born to nonusers, statin use was not associated with neonatal malformations, indicating that statins may be safe to use during pregnancy [23,24,25,26]. Regardless, most of the studies are small and reflect the primarily incidental use of statins. More data regarding the safety of statin use during pregnancy are expected to come out following the new US FDA guideline. Additionally, higher doses of statins have been correlated with an increased risk of toxic effects to the muscles although severe adverse events are considered rare [27,28]. As such, reducing the overall dose of statins for potential anti-ZIKV applications would marginally reduce the risks of adverse effects in both pregnant and nonpregnant users. Herein, we determined whether combinations of lipophilic statins would have synergistic effects against ZIKV infection in vitro to reduce the required dose for treatment.

The combinations that we tested were those of ATO + MEV, ATO + SIM, FLU + MEV, and FLU + SIM. The combinations were chosen based on the form of the statins, where ATO and FLU are active forms and where MEV and SIM are statin prodrugs. The potencies of the statins as cholesterol-lowering drugs corresponded with their potencies as HMGCR inhibitors (Table 5) [29]. As such, in terms of lowering serum cholesterol levels, FLU was considered the least potent statin, SIM was of medium potency, and ATO was of high potency [30]. Meanwhile, MEV, the first statin to be isolated, had not been marketed. Our previous study and this study suggest that the potency of statins as lipid-lowering agents was not translatable to their effectivity against the ZIKV in vitro, as FLU was consistently the most potent statin based on the concentration that reached the maximum inhibition of ZIKV production and infectivity in Vero cells (Table 5). The EC_50_ values obtained in this study were in micromolar concentrations and were much higher than the nanomolar peak (C_max_) and the steady-state serum concentrations (C_ss_) achievable for current ATO and SIM dosing [31,32,33]. However, the micromolar concentrations of FLU in the serum may be achievable in the immediate-release formulation of fluvastatin (fluvastatin IR) [34]. A study also reported a higher C_max_ of FLU among females [35], which may benefit pregnant women who will potentially use FLU against ZIKV infection. Regardless, lowering the overall dose of the statins for clinical applications through combinations would be important. It should be noted that the anti-ZIKV EC_50_ values may vary per cell culture model. Using human cell lines may be more relevant for comparing concentrations in vitro and in clinical applications.

We found that ATO exhibited synergistic effects primarily with MEV against ZIKV infection in vitro. The range of synergistic concentrations were wide and provided room to study more concentration ratios to optimize these synergistic effects. Similarly, FLU was synergistic with MEV and SIM against ZIKV infection in vitro. Higher fractions of MEV appeared to be more beneficial to the FLU + MEV combination, whereas higher fractions of FLU appeared to be more beneficial to the FLU + SIM combination. The use of SIM, a prodrug, has been most correlated with myopathy although this may be a consequence of SIM being a first-line prescription for hyperlipidemia and, thus, may be due to the high frequency of SIM use compared to other statins [36]. On the other hand, FLU is believed to have a lower muscular penetration based on lower reports of rhabdomyolysis [37]. Thus, a combination with higher fractions of FLU to SIM may be safer when administered in vivo and should be considered for the future testing of combinatorial therapies. Additionally, if the anti-ZIKV therapeutic dose for FLU proves to be lower than that applied for lowering cholesterol, then FLU may have lower risks of adverse effects when applied for the treatment of ZIKV infections. The lack of synergistic effects between ATO and SIM may be due to the high binding affinity of ATO to HMGCR. It may be able to fully sequester the HMGCR binding sites, thus blocking the ability of SIM to bind HMGCR. In contrast, the tendency towards synergy of the MEV combinations may be due to non-HMGCR effects. However, because MEV is not marketed, very few exploratory studies outside the HMGCR inhibitory activity of MEV have been performed. Thus, we could not say whether MEV had non-HMGCR targets. However, this would be an interesting topic for future research.

A growing number of in vitro and in vivo evidence suggest the potential of statins as antivirals [38]. Notably, statins have demonstrated activity against other flaviviruses. The intraperitoneal administration of ATO in mouse pups reduced the levels of Japanese encephalitis virus (JEV) nonstructural protein 3 (NS3) and NS5 in the subventricular zones of mouse brains [15]. ATO also abrogated JEV-induced apoptosis and inflammation in neural stem/progenitor cells. These in vivo results may be indicative of the ability of ATO to cross the blood–brain barrier (BBB), corroborating the reported ability of lipophilic statins to cross the BBB [39,40,41]. The study suggested that ATO conferred neuroprotective effects in vivo, which coincides with reports of the neuroprotective effects of statins especially in the context of neurodegenerative diseases [40]. These two properties of statins, the ability to cross the BBB and to protect neurons from damage (whether from apoptosis or inflammation), may help protect the fetus from the neurological complications arising from ZIKV infection.

LOV, another statin that had previously shown activity against the ZIKV in vitro, is the most studied statin against dengue virus infection. It has demonstrated activity in vitro and in vivo [13,14]. In a mouse model, the oral LOV treatment of the mice prior to DENV2 infections reduced viral loads when given at three doses [14]. Meanwhile, the oral LOV treatment of mice post-DENV2 infection required five to six doses to significantly reduce the viral loads. In both pre- and post-DENV infection treatment schemes, the oral LOV treatment did not significantly increase mouse survival. Additionally, in a placebo-controlled clinical trial using LOV (80 mg for 5 days) in adults with confirmed DENV2 infections, LOV was not observed to have significant benefits against DENV2 viremia or against the clinical manifestations of DENV2 infections [42]. It is highly likely that the dose of statins used in both the mouse and human studies were insufficient, which further indicates that drug combinations may be a strategy to reduce the required dose for the treatment of ZIKV infections. Although we previously reported that LOV inhibited the ZIKV, we did not test its combinations with other statins in our current study considering that FLU, which had similar levels of activity, had lower therapeutic concentrations and may also be safer for clinical application.

The pretreatment of human respiratory epithelial cells with FLU has also been reported to reduce SARS-CoV-2 infectivity in vitro, whereas neither SIM nor rosuvastatin (ROS) pretreatment inhibited SARS-CoV-2 infectivity at the tested concentrations [43]. Like ATO, ROS is a high-potency statin; thus, this SARS-CoV-2 study further suggested that the anticholesterol potency of statins was not reflective of its antiviral effects. This study on the effects of statins on SARS-CoV-2-infected cells showed that all statins had similar effects on cholesterol and cholesterol-synthesis-related pathways, suggesting that the effects of FLU on SARS-CoV-2 were independent of its ability to inhibit cholesterol synthesis. Differential proteomic analyses suggested that FLU uniquely affected the protein profile of SARS-CoV-2-infected cells compared to SIM and ROS. Among the proteins uniquely downregulated by FLU are those involved in translation, including polyadenylate-binding protein 1 (PABPC1), eukaryotic translation initiation factor 3 subunit B (EIF3B), ATP-dependent RNA helicase (DDX3X), and IFN-induced dsRNA-dependent serine/threonine-protein kinase (EIF2AK2 or PKR). The translation of the viral RNA strand into proteins is a requirement of RNA virus infections, and these host proteins may be similarly important in ZIKV infections. Notably, PABPC1 has been reported to be utilized by DENV [44]; whether the ZIKV takes advantage of this same protein has not yet been reported. Based on the SARS-CoV-2 study, FLU may have host targets other than HMGCR that contribute to its ability to inhibit viral infections. A docking analysis of statins with the non-Spike proteins of SARS-CoV-2 predicted that, among the statins, FLU would have good binding with the SARS-CoV-2 RNA-dependent RNA polymerase, 3-chymotrypsin-like protease, and helicase [45]. The predicted binding affinity of FLU to the SARS-CoV-2 helicase was particularly high compared to the rest of the statins. However, these interactions have to be verified in vitro. While SARS-CoV-2 and the ZIKV are from different families, they are both RNA viruses. Viral proteins involved in the replication and translation machineries of RNA viruses have conserved regions which have made the broad-spectrum activity of certain antivirals possible. It is probable that FLU binds one or two similar proteins that the ZIKV genome encodes, and this would be worth investigating as a potential target.

ATO has similarly been reported to inhibit SARS-CoV-2 infection in vitro [46]. However, the study did not compare the effects of the statins, so we could not correlate the effects of ATO with its effects on cholesterol or HMGCR. On the other hand, a population-based case-control study in South Korea in 2020 showed that ATO was significantly associated with reduced COVID-19 severity and mortality [47]. No such effects were observed for ROS and other statins. Although the SARS-CoV-2 studies on statins do not directly translate to the ZIKV, they illustrate that the pleiotropic (noncholesterol) effects of the statins are not equal. They may, in fact, be independent of HMGCR inhibition. Thus, when it comes to evaluating statins for their antiviral effects, the statins should not be treated as a single class but rather as individual drugs. In vitro studies should therefore refrain from using only drug class representatives for evaluation, and clinical studies should be able to group patient subjects into separate statin groups for better comparisons of statin effectivity.

The differences in the biochemical characteristics of the statins, including lipophilicity, cell membrane penetration, etc., may underlie the differences in the statin potencies as antivirals, at least in vitro [48,49]. The aforementioned studies illustrated that certain statins may have HMGCR-independent targets. If FLU indeed has targets in the translation machinery or on the virus, then its effects on translation or the virus may act in tandem with its ability to inhibit HMGCR. This may then explain why FLU appears to be superior to other statins in the context of ZIKV and SARS-CoV-2 infection. In the context of the statin combinations, the possibility of a non-HMGCR binding target for FLU or another statin would be favorable. If this were the case, although two statins would compete for the HMGCR active site, the binding of FLU to another target would sequester it from HMGCR. This then would allow another statin to come in to inhibit HMGCR. FLU would then act on its other target and on some free HMGCR molecules, which may raise the overall effectivity of the combination. We previously showed that most statins did not reduce ZIKV production to the same extent in vitro; treatment with SIM, MEV, ATO, and FLU over 4 days reduced ZIKV production in Vero cells by one, two, and three logarithmic values, respectively. Potentially, utilizing two statins with different targets involved in ZIKV replication may increase the overall effectivity of the statin treatment and may have larger effects on virus production. The investigation of the effects of statin combinations on ZIKV growth kinetics would verify this hypothesis.

Statins are often used with other drugs for high blood pressure. Studies on combining statins with amlodipine, ezetimibe, and fenofibrate have reported no significantly increased incidence of adverse effects [50,51,52]. However, since the statin–statin combinations were not used for lipid-lowering (i.e., one statin was often discontinued before another was prescribed), we could not conclude whether the statin combinations at the current doses would be safe. However, potential antiviral applications would be of shorter durations than the antilipidemic application of statins and may pose lower risks for muscle-related side effects. Instead, the safety of statins and/or statin combinations for fetuses and pregnant women are of the utmost concern and should be closely evaluated.

Here, we have shown that the active statins ATO and FLU exhibited synergistic tendencies when used with the statin prodrugs MEV and SIM against ZIKV infection in vitro. The ATO/FLU combinations with MEV were more likely to be synergistic than the combinations with SIM. However, FLU may have been synergistic with SIM when the combination had higher fractions of FLU. Our results suggested that the overall therapeutic dose of the statins against the ZIKV and the dose-related adverse effects could potentially be lowered through combinatorial therapy. However, our study was limited to one cell culture model. More combinatorial assays of statins using other in vitro and in vivo models should be performed to determine the clinical applicability and safety of these statins for anti-ZIKV applications. Combinations of statins with other anti-ZIKV candidates should also be considered to target more stages in the ZIKV replication cycle.

## Figures and Tables

**Table 1:** Half-maximal inhibitory concentrations of the atorvastatin and mevastatin combinations.

Combi.	Starting Conc. (µM)		EC_50_ *^a^*		FIC_50_ *^b^*		CI
(Ratio)	ATO	MEV	ATO (nM)	MEV (nM)	ATO	MEV	ΣFIC_50_ *^b^*
C1 (5:0)	8	0	4568 (4102–5034)	0	1.00	0	1.00
C2 (4:1)	6.4	2.4	3433 (3312–3554)	1287 (1237–1337)	0.750 (±0.003)	0.378 (±0.007)	1.129 (±0.011)
C3 (3:2)	4.8	4.8	1837 (1770–1908)	1837 (1764–1916)	0.402 (±0.008)	0.541 (±0.019)	0.943 (±0.027)
C4 (2:3)	3.2	7.2	971.7 (899–1044)	2186 (2917–2355)	0.212 (±0.002)	0.642 (±0.017)	0.854 (±0.019)
C5 (1:4)	1.6	9.6	456.6 (412–501)	2739 (2474–3004)	0.100 (±0.002)	0.805 (±0.0001)	0.905 (±0.001)
C6 (0:5)	0	12	0	3404 (3020–3788)	0	1.00	1.00

Abbreviations: Combi.—combination; conc.—concentration; ATO—atorvastatin; MEV—mevastatin; EC_50_—half-maximal effective concentration; FIC_50_—fractional inhibitory concentration with 50% effectivity; and CI—combinatorial index (the sum of the FIC_50_ of the two drugs). *^a^* EC_50_ values are expressed with 95% confidence intervals. *^b^* FIC_50_/CI values are expressed with ± standard error of the mean.

**Table 2:** Half-maximal inhibitory concentrations of the atorvastatin and simvastatin combinations.

Combi.	Starting Conc. (µM)		EC_50_ *^a^*		FIC_50_ *^b^*		CI
(Ratio)	ATO	SIM	ATO (nM)	SIM (nM)	ATO	SIM	ΣFIC_50_ *^b^*
C1 (5:0)	8	0	4639 (3274–6574)	0	1.00	0	1.00
C2 (4:1)	6.4	0.8	2391 (2257–2532)	298.8 (282–317)	0.703 (±0.163)	0.228 (±0.027)	0.930 (±−0.136)
C3 (3:2)	4.8	1.6	1870 (1741–2009)	623.5 (581–670)	0.524 (±0.136)	0.448 (±0.040)	0.972 (±0.096)
C4 (2:3)	3.2	2.4	1138 (973–1330)	852.5 (738–985)	0.327 (±0.110)	0.609 (±0.003)	0.934 (±0.106)
C5 (1:4)	1.6	3.2	536.8 (446–644)	1068 (907–1258)	0.270 (±0.162)	0.808 (±0.006)	1.08 (±0.156)
C6 (0:5)	0	4	0	1804 (1336–2435)	0	1.00	1.00

Abbreviations: Combi.—combination; conc.—concentration; ATO—atorvastatin; SIM—simvastatin; EC_50_—half-maximal effective concentration; FIC_50_—fractional inhibitory concentration with 50% effectivity; and CI—combinatorial index (the sum of the FIC_50_ of the two drugs). *^a^* EC_50_ values are expressed with 95% confidence intervals. *^b^* FIC_50_/CI values are expressed with ± standard error of the mean.

**Table 3:** Half-maximal inhibitory concentrations of the fluvastatin and mevastatin combinations.

Combi.	Starting Conc. (µM)		EC_50_ *^a^*		FIC_50_ *^b^*		CI
(Ratio)	FLU	MEV	FLU (nM)	MEV (nM)	FLU	MEV	ΣFIC_50_ *^b^*
C1 (5:0)	3	0	1726 (1544–1978)	0	1.00	0	1.00
C2 (4:1)	2.4	2.4	1128 (992–1266)	1128 (992–1266)	0.684 (±0.051)	0.371 (±0.118)	1.054 (±0.169)
C3 (3:2)	1.8	4.8	641.5 (911–1044)	1711 (1498–1952)	0.367 (±0.075)	0.522 (±0.005)	0.889 (±0.080)
C4 (2:3)	1.2	7.2	641.9 (562–732.2)	1952 (1651–2319)	0.213 (±0.066)	0.642 (±0.055)	0.855 (±0.121)
C5 (1:4)	0.6	9.6	325.3 (275–387)	3053 (2513–3656)	0.0.99 (±0.049)	0.740 (±0.215)	0.839 (±0.264)
C6 (0:5)	0	12	0	3096 (2726–3519)	0	1.00	1.00

Abbreviations: Combi.—combination; conc.—concentration; FLU—fluvastatin; MEV—mevastatin; EC_50_—half-maximal effective concentration; FIC_50_—fractional inhibitory concentration with 50% effectivity; and CI—combinatorial index (the sum of the FIC_50_ of the two drugs). *^a^* EC_50_ values are expressed with 95% confidence intervals. *^b^* FIC_50_/CI values are expressed with ± standard error of the mean.

**Table 4:** Half-maximal inhibitory concentrations for the fluvastatin and simvastatin combinations.

Combi.	Starting Conc. (µM)		EC_50_ *^a^*		FIC_50_ *^b^*		CI
(Ratio)	FLU	SIM	FLU (nM)	SIM (nM)	FLU	SIM	ΣFIC_50_ *^b^*
C1 (5:0)	3	0	1630 (1513–1752)	0	1.00	0	1.00
C2 (4:1)	2.4	0.8	1073 (995–1154)	357.8 (331–385)	0.664 (±0.056)	0.174 (±0.026)	0.838 (±0.082)
C3 (3:2)	1.8	1.6	966.5 (911–1044)	859.1 (810–928)	0.582 (±0.053)	0.373 (±0.027)	0.955 (±0.080)
C4 (2:3)	1.2	2.4	641.9 (601–684)	1284 (1202–1368)	0.393 (±0.022)	0.567 (±0.022)	0.956 (±0.043)
C5 (1:4)	0.6	3.2	338.1 (315–361)	1803 (1681–1925)	0.208 (±0.004)	0.802 (±0.003)	1.01 (±0.007)
C6 (0:5)	0	4	0	2259 (2091–2427)	0	1.00	1.00

Abbreviations: Combi.—combination; conc.—concentration; FLU—fluvastatin; SIM—simvastatin; EC_50_—half-maximal effective concentration; FIC_50_—fractional inhibitory concentration with 50% effectivity; and CI—combinatorial index (the sum of the FIC_50_ of the two drugs). *^a^* EC_50_ values are expressed with 95% confidence intervals. *^b^* FIC_50_/CI values are expressed with ± standard error of the mean.

**Table 5:** Comparison of effective in vitro concentrations and serum concentrations of statins.

Statin	EC_50_ (µM)	C_max_	C_ss_	HMGCR IC_50_ (nM)
Atorvastatin	4.6	8–40 nM	31.9 ng/mL (57.10 nM)	8.2
Fluvastatin	1.7	40 mg dose: 1.08 µM in males 1.58 µM in females	Lescol XL, 80 mg: 7.71–102.4 ng/mL (18–249 nM) Lescol XL, 160 mg: 25.3–258.8 (61–629 nM) Fluvastatin IR, 40 mg: 9.19–442.8 ng/mL (22–1077 nM)	27.6
Mevastatin	3.4	-	-	23
Simvastatin	2.3	19–31 nM	1.68 ng/mL (4.01 nM)	11.2

C_max,_ or peak plasma concentrations, and Css, or steady-state serum concentrations, were derived from [31,32,33,34,35]. HMGCR half-maximal inhibitory concentrations (IC_50_) were cell-free values based on [29].

## Data Availability

Data are contained within the article and Supplementary Materials.

## Supplementary Materials

The following supporting information can be downloaded at https://www.mdpi.com/article/10.3390/pharmaceutics15010050/s1, Figure S1: Effects of the combination of atorvastatin (ATO) with (a) mevastatin (MEV) and (b) simvastatin (SIM) on Vero cell viability; Figure S2: Effects of the combination of fluvastatin (FLU) with (a) mevastatin (MEV) and (b) simvastatin (SIM) on Vero cell viability.
