# Supplementary material for: Effects of Statin Combinations on Zika Virus Infection in Vero Cells
Source: Pharmaceutics. 2022 Dec 23;15(1):50. doi: 10.3390/pharmaceutics15010050 (PMC9864436; doi:10.3390/pharmaceutics15010050)
Supplement: Supplementary file 1 [file pharmaceutics-15-00050-s001.zip › pharmaceutics-2034396-supplementary.pdf]

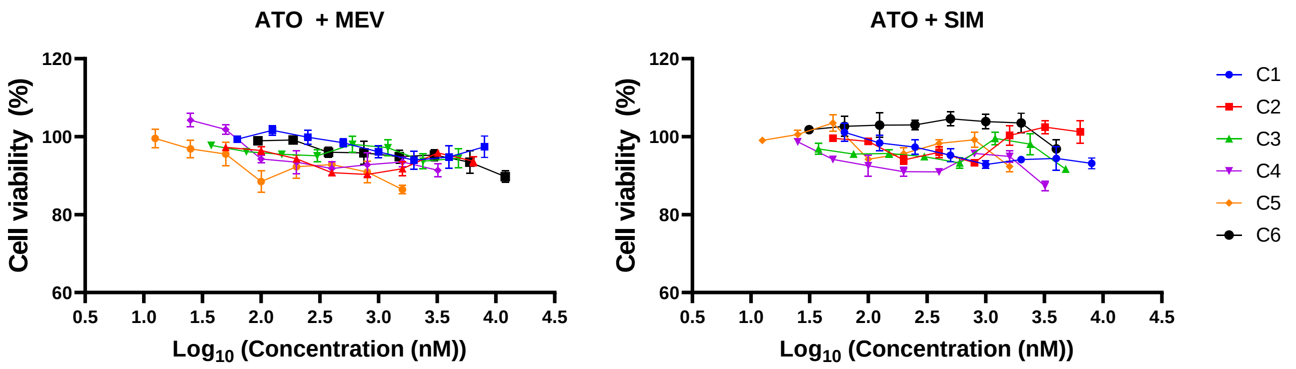

(a) (b)

Figure S1. Effects of the combination of atorvastatin (ATO) with (**a**) mevastatin (MEV) and (**b**) simvastatin (SIM) on Vero cell viability. Uninfected Vero cells were treated with different drug combinations (n = 3 per combination/dilution). Cell viabilities were calculated relative to that of the DMSO (0.5%) uninfected control, which was assigned as 100%. All concentrations for C1–C5 are based on ATO concentrations. C6 concentrations reflect the concentrations of (**a**) MEV and (**b**) SIM in C6.

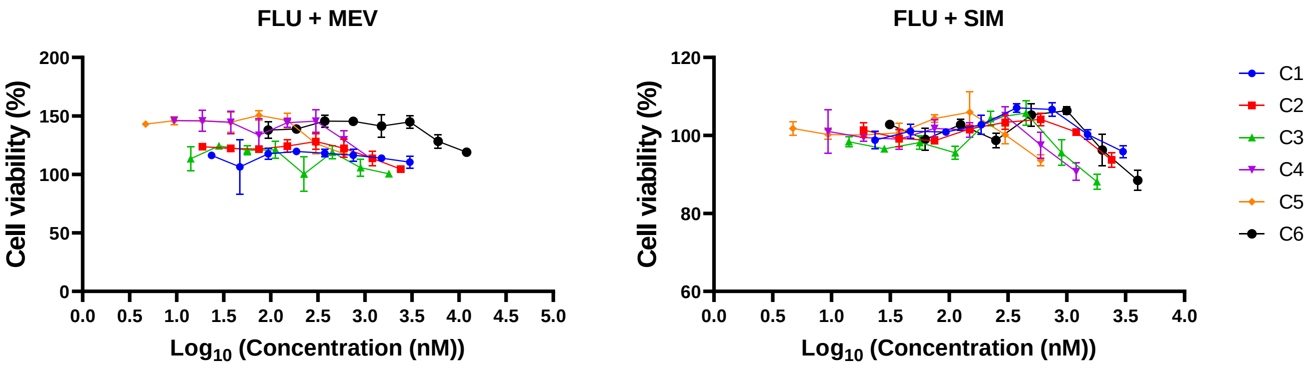

(a) (b)

Figure S2. Effects of the combination of fluvastatin (FLU) with (**a**) mevastatin (MEV) and (**b**) simvastatin (SIM) on Vero cell viability. Uninfected Vero cells were treated with different drug combinations (n = 3 per combination/dilution). Cell viabilities were calculated relative to that of the DMSO (0.5%) uninfected control, which was assigned as 100%. All concentrations for C1–C5 are based on FLU concentrations. C6 concentrations reflect the concentrations of (**a**) MEV and (**b**) SIM in C6.
